# Individual and combinatorial effects of SNP and NaHS on morpho-physio-biochemical attributes and phytoextraction of chromium through Cr-stressed spinach (*Spinacia oleracea* L.)

**DOI:** 10.3389/fpls.2022.973740

**Published:** 2022-08-17

**Authors:** Jing Ma, Muhammad Hamzah Saleem, Ghulam Yasin, Sahar Mumtaz, Freeha Fatima Qureshi, Baber Ali, Sezai Ercisli, Sadeq K. Alhag, Ahmed Ezzat Ahmed, Dan C. Vodnar, Iqbal Hussain, Romina Alina Marc, Fu Chen

**Affiliations:** ^1^School of Public Administration, Hohai University, Nanjing, China; ^2^College of Plant Science and Technology, Huazhong Agricultural University, Wuhan, China; ^3^Institute of Botany, Bahauddin Zakariya University, Multan, Pakistan; ^4^Division of Science and Technology, Department of Botany, University of Education, Lahore, Pakistan; ^5^Department of Botany, Government College University Faisalabad, Faisalabad, Pakistan; ^6^Department of Plant Sciences, Quaid-i-Azam University, Islamabad, Pakistan; ^7^Department of Horticulture Faculty of Agriculture Ataturk University, Erzurum, Turkey; ^8^Biology Department, College of Science and Arts, King Khalid University, Muhayil, Saudi Arabia; ^9^Biology Department, College of Science, Ibb University, Ibb, Yemen; ^10^Department of Biology, College of Science, King Khalid University, Abha, Saudi Arabia; ^11^Department of Theriogenology, Faculty of Veterinary Medicine, South Valley University, Qena, Egypt; ^12^Institute of Life Sciences, Faculty of Food Science and Technology, University of Agricultural Sciences and Veterinary Medicine, Cluj-Napoca, Romania; ^13^Food Engineering Department, Faculty of Food Science and Technology, University of Agricultural Science and Veterinary Medicine Cluj-Napoca, Cluj-Napoca, Romania

**Keywords:** heavy metal, staple food, chemical compounds, antioxidant machinery, essential nutrients

## Abstract

Chromium (Cr) is a toxic heavy metal that contaminates soil and water resources after its discharge from different industries. A pot experiment was conducted to determine the effects of single and/or combined application of sodium nitroprusside (SNP) (250 μM) and sodium hydrogen sulfide (NaHS) (1 mM) on growth, photosynthetic pigments, gas exchange characteristics, oxidative stress biomarkers, antioxidant machinery (enzymatic and non-enzymatic antioxidants), ion uptake, organic acid exudation, and Cr uptake of spinach (*Spinacia oleracea* L.) exposed to severe Cr stress [Cr: 0 (no Cr), 150, and 300 μM]. Our results depicted that Cr addition to the soil significantly (*P* < 0.05) decreased plant growth and biomass, gas exchange attributes, and mineral uptake by *S*. *oleracea* when compared to the plants grown without the addition of Cr. However, Cr toxicity boosted the production of reactive oxygen species (ROS) by increasing the content of malondialdehyde (MDA), which is the indication of oxidative stress in *S*. *oleracea*, and was also manifested by hydrogen peroxide (H_2_O_2_) content and electrolyte leakage to the membrane-bound organelles. The results showed that the activities of various antioxidative enzymes, such as superoxidase dismutase (SOD), peroxidase (POD), catalase (CAT), and ascorbate peroxidase (APX), and the content of non-enzymatic antioxidants, such as phenolic, flavonoid, ascorbic acid, and anthocyanin, initially increased with an increase in the Cr concentration in the soil. The results also revealed that the levels of soluble sugar, reducing sugar, and non-reducing sugar were decreased in plants grown under elevating Cr levels, but the accumulation of the metal in the roots and shoots of *S*. *oleracea*, was found to be increased, and the values of bioaccumulation factor were <1 in all the Cr treatments. The negative impacts of Cr injury were reduced by the application of SNP and NaHS (individually or combined), which increased plant growth and biomass, improved photosynthetic apparatus, antioxidant enzymes, and mineral uptake, as well as diminished the exudation of organic acids and oxidative stress indicators in roots of *S*. *oleracea* by decreasing Cr toxicity. Here, we conclude that the application of SNP and NaHS under the exposure to Cr stress significantly improved plant growth and biomass, photosynthetic pigments, and gas exchange characteristics; regulated antioxidant defense system and essential nutrient uptake; and balanced organic acid exudation pattern in *S*. *oleracea*.

## Introduction

The contamination of soil with heavy metals is a severe worldwide issue due to the negative impacts of heavy metals on environmental safety (Ashraf et al., 2022; Zaheer et al., 2022). The higher accumulation of heavy metals in the environment through various activities, such as smelting, fossil fuel burning, disposal of industrial products (paints, enamels, alloys, steels, varnishes, and inks), mining, and excessive fertilization, is of greater concern to the ecosystem and public health due to their enhanced tendency of bioaccumulation and persistence (Gill et al., 2013; Heile et al., 2021; Javed et al., 2021). Heavy metals are of high ecological significance due to their persistence, toxicity, and bioaccumulation capacity, and also the excess amount of heavy metals in soil significantly reduces plant production and yield (Amna et al., 2021; Khattak et al., 2021). Among various heavy metals, chromium (Cr) is an extremely noxious metal to living organisms, and negative effects have been reported in humans (Shahid et al., 2017), animals (Ugwu and Agunwamba, 2020), plants (Ertani et al., 2017), and micro-organisms (Ranieri et al., 2020). Cr is a major toxic element discharged into the environment through various industries, such as tanning, electroplating, manufacturing of pigments, production of nuclear weapons, and corrosion control (Zaheer et al., 2020b; Hussain et al., 2021). This extensive industrial use of Cr composites and their subsequent release, without prior treatment, into the surrounding environment contaminates the entire ecosystem and can lead to catastrophic health risks (Zaheer et al., 2020c; Zainab et al., 2021). Hence, it is immensely required to safeguard the plants from Cr toxicity to counter the phytotoxicity and oxidative stress triggered by the uptake of Cr in plants.

Therefore, the accumulation of Cr by plants and eventually its presence in foodstuffs must be eradicated. However, vegetables like spinach (*Spinacia oleracea* L.) showed more tolerance to Cr stress when irrigated on Cr-contaminated soil (Sehrish et al., 2019; Zaheer et al., 2020a; Hussain et al., 2021). *Spinacia oleracea* is a worldwide cultivated vegetable crop because of its relatively higher growth rates, increased production of biomass, and use of heavy metals and other important soil nutrients (Agarwal et al., 2018). Furthermore, *S. oleracea* has been extensively investigated on the basis of these distinctive features to analyze its growth performance and stress responses to various heavy metals (Shahid et al., 2020; Saleem et al., 2022a). Among different gaseous compounds, exogenous use of nitric oxide (NO) has also gained more importance. Nitric oxide in the form of SNP (donor of NO) acts as a free radical and is lipophilic and volatile in nature (Panda et al., 2011). Apart from its regulatory roles in plants, such as improving seed germination and seedling growth (Ali et al., 2017), it also plays a protective role against different abiotic stresses, including metal toxicity, temperature, and drought (Amooaghaie and Roohollahi, 2017; Ozfidan-Konakci et al., 2020; Habib et al., 2021). NaHS (donor of H_2_S) is a colorless soluble gas that can be toxic. However, during the last decade, NaHS has been recognized as a vital molecular signal in plants (Ozfidan-Konakci et al., 2020; Mfarrej et al., 2021). For instance, NaHS maintains the membrane integrity of *Triticum aestivum* root tips under Cu toxicity, reduces chlorophyll degradation, and prevents oxidative damage (Ali et al., 2022a). NaHS also promotes photosynthesis in *Hordeum vulgare* under aluminum toxicity (Delhaize et al., 2009). NaHS regulates different developmental processes, such as lateral root and adventitious root formation and germination (Kaya et al., 2020a). Therefore, NO has a significant ability to mediate plant responses to environmental stresses.

The primary objective of this study was to assess the effect of exogenous NaHS and SNP (individual or combined) on growth, photosynthetic pigments, gas exchange characteristics, oxidative stress biomarkers, antioxidant machinery (enzymatic and non-enzymatic antioxidants), ion uptake, organic acid exudation, and Cr uptake in spinach (*Spinacia oleracea* L.) under Cr stress. Furthermore, we investigated the efficiency of exogenous NaHS and SNP (individual or combined) administration in plant defense response during Cr stress. *S. oleracea* is a staple food in many regions of the world and is highly sensitive to Cr stress. We hypothesized that exogenous application of these signaling molecules may improve growth and grain yield in *S. oleracea* through modulation of key physiochemical processes under Cr stress. Therefore, this study aimed to ascertain the effect of NaHS and SNP (individual or combined) on growth, photosynthetic pigments, gas exchange characteristics, oxidative stress biomarkers, antioxidant machinery (enzymatic and non-enzymatic antioxidants), ion uptake, organic acid exudation, and Cr uptake in *S. oleracea*, grown under different levels of Cr-spiked soil.

## Materials and methods

### Experimental setup

The mature and healthy seeds of spinach (*Spinacia oleracea* L.) [Local Sindhi (a native variety and most commonly used in Pakistan)] were collected from the Ayub Agricultural Research Institute (AARI), Faisalabad 38000, Pakistan. Previously, *S. oleracea* has been grown as a potential food crop under Cr-polluted soil (Zaheer et al., 2020d; Hussain et al., 2021). A pot experiment was conducted in the greenhouse of the Department of Botany, Government College University, Faisalabad 38000, Punjab, Pakistan (31° 24/N, 73° 04/E). Before sowing, the seeds were carefully washed and sterilized in 0.1% HgCl_2_ solution for 1 min and then washed three times with distilled water. All pots (35 cm height × 25 cm width) were covered with plastic bags, with each containing 5 kg of uncontaminated soil. For the complete removal of cations and anions, the soil was washed with distilled water several times. After that, in each pot, about 10 seeds were sown, and each pot was kept in a greenhouse where they received natural light and air. Each pot was placed in a randomized manner with four replicates per treatment being carried out with five plants in each pot. The total duration of experimental treatments was 2 months under controlled conditions where they received natural light with day/night temperature of 35/40°C and day/ night humidity of 60/70%. After 1 week, the propagated *S. oleracea* seedlings were treated by inducing Cr stress. All the pots were categorized into three groups: (I) without any Cr treatment, (II) addition of 150 μM of Cr, and (III) addition of 300 μM of Cr. Before sowing the plants, Cr was artificially supplied using potassium dichromate (K_2_Cr_2_O_7_) at various concentrations. The concentrations of Cr used in this study were higher than those used in our previously conducted experiment on *S. oleracea* (Hussain et al., 2021). After adding Cr, the pots were equilibrated for 2 months with four cycles of saturation with distilled water and air drying. Foliar sodium hydrogen sulfide (NaHS; 1 mM) and sodium nitroprusside (SNP; 250 μM) were administered for 1 week following the imposition of Cr stress in the soil. The levels of SNP and NaHS were selected on the basis of our previous study on wheat (*Triticum aestivum* L.) (Mfarrej et al., 2021). As a control, plants were also sprayed with distilled water. The soil used for this experiment was collected from the experimental station of Government College University, Faisalabad, and its properties are given in Supplementary Table 1. The soil sample was air dried, passed through a 5-mm sieve, and was water saturated two times before being used in pots. Irrigation with Cr-free water and other intercultural operations were performed when needed.

### Sampling and data collection

After 2 months, three seedlings were uprooted and washed gently with the help of distilled water to eliminate the aerial dust and deposition. All plants were harvested on 1st October 2021 for the analysis of various morphological parameters. Leaves and root samples from each treatment group were picked after 1 month for chlorophyll, carotenoid, and antioxidant analysis. The leaves were washed with distilled water, placed in liquid nitrogen, and stored at −80°C for further analysis. The plants from each treatment were washed with tap water to remove debris and waste and then with distilled water. Plant length (shoot and root) was measured using a measuring scale. The number of leaves was measured by simple counting, and leaf area was also measured. The fresh weight (shoot and root) of the plant was determined by measuring the weight of the plant with a digital weighing balance. Later, the root and shoot were dried in an oven at 105°C for 1 h, and then at 70°C for 72 h to determine their dry weight. Roots were immersed in 20 mM Na2EDTA for 15–20 min to remove Cr adhered to the surface of the roots. Then, the roots were washed three times with distilled water and finally once with deionized water and dried for further analysis. Although this experiment was conducted in pots, for the collection of organic acids, two seedlings were transferred to the rhizoboxes which consisted of a plastic sheet, nylon net, and wet soil (Javed et al., 2013). After 48 h, plants were taken from the rhizoboxes, and the roots were washed with redistilled water to collect the exudates from the root surface. The samples were filtered through a 0.45-μm filter (MillexHA, Millipore) and collected in Eppendorf tubes (Greger and Landberg, 2008). The collected samples were mixed with NaOH (0.01 M) to analyze the organic acids. However, the samples used for the analysis of oxalic acid were not treated with NaOH (Javed et al., 2013).

### Determination of photosynthetic pigments and gas exchange characteristics

Leaves were collected for the determination of chlorophyll and carotenoid contents. For chlorophylls, 0.1 g of fresh leaf sample was extracted with 8 mL of 95% acetone for 24 h at 4°C in the dark. The absorbance was measured by a spectrophotometer (UV-2550; Shimadzu, Kyoto, Japan) at 646.6, 663.6, and 450 nm. Chlorophyll content was calculated by the standard method described previously (Arnon, 1949).

Net photosynthesis (*Pn*), leaf stomatal conductance *(Gs)*, transpiration rate (*Ts*), and intercellular carbon dioxide concentration (*Ci*) were measured from four different plants in each treatment group. Measurements were conducted between 11:30 and 13:30 on days with a clear sky. Rates of leaf *Pn, Gs, Ts*, and *Ci* were measured with an LI-COR gas-exchange system (LI-6400; LI-COR Biosciences, Lincoln, NE, USA) with a red-blue LED light source on the leaf chamber. In the LI-COR cuvette, CO_2_ concentration was set as 380 mmol mol^−1^, and LED light intensity was set at 1,000 mmol m^−2^ s^−1^, which was the average saturation intensity for photosynthesis in *S. olercea* (Zaheer et al., 2022).

### Determination of oxidative stress indicators

The degree of lipid peroxidation was evaluated as malondialdehyde (MDA) content. Briefly, 0.1 g of frozen leaves was ground at 4°C in a mortar with 25 mL of 50 mM phosphate buffer solution (pH 7.8) containing 1% polyethene pyrrole. The homogenate was centrifuged at 10,000 × *g* at 4°C for 15 min. The mixtures were heated at 100°C for 15–30 min and then quickly cooled in an ice bath. The absorbance of the supernatant was recorded by using a spectrophotometer (xMark™ Microplate Absorbance Spectrophotometer; Bio-Rad, United States) at wavelengths of 532, 600, and 450 nm. Lipid peroxidation was expressed as l mol g^−1^ by using the formula: 6.45 (A532-A600)-0.56 A450. Lipid peroxidation was measured by using a method published previously (Heath and Packer, 1968).

To estimate the content of H_2_O_2_ in plant tissues (root and leaf), 3 mL of sample extract was mixed with 1 mL of 0.1% titanium sulfate in 20% (v/v) H_2_SO_4_ and centrifuged at 6,000 × *g* for 15 min. The intensity of the yellow color was evaluated at 410 nm. The H_2_O_2_ level was computed by the extinction coefficient of 0.28 mmol^−1^ cm^−1^. The content of H_2_O_2_ was measured by the method presented previously (Jana and Choudhuri, 1981).

Stress-induced electrolyte leakage (EL) of the uppermost stretched leaves was determined by using the methodology of Dionisio-Sese and Tobita (1998). The leaves were cut into minor slices (5 mm length) and placed in test tubes containing 8 mL of distilled water. These tubes were incubated and transferred to a water bath for 2 h prior to measuring the initial electrical conductivity (EC_1_). The samples were autoclaved at 121°C for 20 min and then cooled down to 25°C before measuring the final electrical conductivity (EC_2_). Electrolyte leakage was calculated by the following formula:


$$\begin{array}{l} \text{EL}=\left(\text{E}{\text{C}}_{1}/\text{E}{\text{C}}_{2}\right)\times 100 \end{array}$$

### Determination of antioxidant enzyme activities

To evaluate enzyme activities, fresh leaves (0.5 g) were homogenized in liquid nitrogen, and 5 mL of 50 mmol sodium phosphate buffer (pH 7.0), including 0.5 mmol EDTA and 0.15 mol NaCl, was added. The homogenate was centrifuged at 12,000 × *g* for 10 min at 4°C, and the supernatant was used for measuring superoxidase dismutase (SOD) and peroxidase (POD) activities. SOD activity was assayed in 3 mL of reaction mixture containing 50 mM sodium phosphate buffer (pH 7), 56 mM nitro blue tetrazolium, 1.17 mM riboflavin, 10 mM methionine, and 100 μL of enzyme extract. Finally, the sample was measured by using a spectrophotometer (xMark™ Microplate Absorbance Spectrophotometer; Bio-Rad). Enzyme activity was measured by using a previously established method (Chen and Pan, 1996) and expressed as U g^−1^ FW.

The POD activity in the leaves was estimated based on a previous method (Sakharov and Ardila, 1999) by using guaiacol as the substrate. A reaction mixture (3 mL) containing 0.05 mL of enzyme extract, 2.75 mL of 50 mM phosphate buffer (pH 7.0), 0.1 mL of 1% H_2_O_2_, and 0.1 mL of 4% guaiacol solution was prepared. An increase in the absorbance at 470 nm due to guaiacol oxidation was recorded for 2 min. One unit of enzyme activity was defined as the amount of the enzyme.

Catalase (CAT) activity was analyzed according to a previous method (Aebi, 1984). The assay mixture (3.0 mL) was composed of 100 μL of enzyme extract, 100 μL of H_2_O_2_ (300 mM), and 2.8 mL of 50 mM phosphate buffer with 2 mM ETDA (pH 7.0). The CAT activity was measured based on the decline in absorbance at 240 nm due to the loss of H_2_O_2_ (ε = 39.4 mM^−1^ cm^−1^).

Ascorbate peroxidase (APX) activity was measured according to a previous method (Nakano and Asada, 1981). The mixture containing 100 μL of enzyme extract, 100 μL of ascorbate (7.5 mM), 100 μL of H_2_O_2_ (300 mM), and 2.7 mL of 25 mM potassium phosphate buffer with 2 mM EDTA (pH 7.0) was used for measuring APX activity. The oxidation pattern of ascorbate was estimated based on the variations in wavelength at 290 nm (ε = 2.8 mM^−1^ cm^−1^).

### Determination of non-enzymatic antioxidant, sugar, and proline content

The ethanol extracts of the plant were prepared for the determination of non-enzymatic antioxidants and some key osmolytes. For this purpose, 50 mg of dry plant material was homogenized with 10 mL of ethanol (80%) and filtered through Whatman No. 41 filter paper. The residue was re-extracted with ethanol, and the two extracts were pooled together to a final volume of 20 mL. The content of flavonoids (Pekal and Pyrzynska, 2014), phenolics (Ali et al., 2022b), ascorbic acid (Azuma et al., 1999), anthocyanin (Lewis et al., 1998), total sugars (Dubois et al., 1956), and free amino acids was determined in the extracts.

Fresh leaf material (0.1 g) was mixed thoroughly in 5 mL of aqueous sulfosalicylic acid (3%). The mixture was centrifuged at 10,000 × *g* for 15 min, and an aliquot (1 mL) was poured into a test tube containing 1 mL of acidic ninhydrin and 1 mL of glacial acetic acid. The reaction mixture was first heated at 100°C for 10 min and then cooled in an ice bath. The reaction mixture was extracted with 4 mL of toluene, and the test tubes were vortexed for 20 s and cooled. Thereafter, the light absorbance at 520 nm was measured by using a UV–VIS spectrophotometer (Hitachi U-2910, Tokyo, Japan). The free proline content was determined on the basis of the standard curve at 520 nm absorbance and expressed as μmol (g FW) ^−1^ (Bates et al., 1973).

### Determination of nutrient content

For nutrient analysis, plant roots and shoots were washed two times in redistilled water, dipped in 20 mM EDTA for 3 s, and then, again, washed with deionized water two times for the removal of adsorbed metal on the plant surface. The washed samples were then oven-dried for 24 h at 105°C. The dried roots and shoots were digested by using a wet digestion method in HNO_3_: HClO_4_ (7:3, v/v) until clear samples were obtained. Each sample was filtered and diluted with redistilled water up to 50 mL. The contents of Fe, Ca, Mg, and P in the roots and shoots were analyzed by using the atomic absorption spectrophotometer (AAS) model Agilent 240FS-AA.

### Root exudate analysis and determination of Cr concentration

In order to determine the concentration of organic acids, freeze-dried exudates were mixed with ethanol (80%), and 20 μL of the solutions were injected into the C18 column (Brownlee Analytical C-183 μm; length 150 × 4.6 mm, USA). Quantitative analysis of organic acids in root exudates was executed with high-performance liquid chromatography (HPLC), having a Flexer FX-10 UHPLC isocratic pump (PerkinElmer, MA, USA). The mobile phase used in HPLC comprised an acidic solution of acetonitrile containing acetonitrile:H_2_SO_4_: acetic acid in ratios of 15:4:1, respectively, and pH of 4.9. The samples were analyzed at a flow rate of 1.0 mL min^−1^ for a time period of 10 min. The inner temperature of the column was fixed at 45°C, and quantification of organic acids was carried out at 214 nm wavelength with the help of a detector (UV–VIS Series 200, USA) as described previously (UdDin et al., 2015). Freeze-dried samples were dissolved in redistilled water, and the pH of the exudates was recorded with LL micro-pH glass electrode by using a pH meter (ISTEK Model 4005–08007 Seoul, South Korea).

Plant samples were vigilantly digested via the di-acid (HNO_3_-HClO_4_) technique. About 0.5 g of dry sample of roots and shoots of the plants was taken into the flask containing 10 mL of HNO3- HClO_4_ (3:1, v:v); this mixture was then retained overnight. The final digestion of these plant samples was completed after the addition of HNO_3_ (5 mL) and then placed on the hot plate for complete digestion as described previously (Rehman et al., 2015). Atomic absorption spectrophotometer (AAS) was used to determine the exact amount of Cr present in the shoots and roots of the plant.

The bioaccumulation factor (BAF) was calculated as the ratio of the Cr concentration in the tissues and the Cr concentration in the soil using the following formula:


$$\begin{array}{lll} \text{BAF } & = & \text{ Cr concentration in plant tissues}/\text{Cr concentration}\\ \text{ in soil} \end{array}$$

### Statistical analysis

The normality of data was analyzed using IBM SPSS software (Version 21.0. Armonk, NY, USA: IBM Corp) through a multivariate *post-hoc* test, followed by Duncan's test in order to determine the interaction among significant values. Thus, the differences between treatments were determined by using ANOVA, and the least significant difference test (*P* < 0.05) was used for multiple comparisons between treatment mean values where significant. Tukey's HSD *post-hoc* test was used to compare the multiple comparisons of mean values. The analysis showed that the data in this study were almost normally distributed. The graphical presentation was carried out using Origin-Pro 2019. The Pearson correlation coefficients between the measured variables of *S. oleracea* were also calculated. The plots of principal component analysis on *S. oleracea* parameters were carried out using the RStudio software.

## Results

### Impact of individual/or combinatorial application of SNP and NaHS on plant growth and photosynthetic pigments under Cr stress

**Figure 1:**
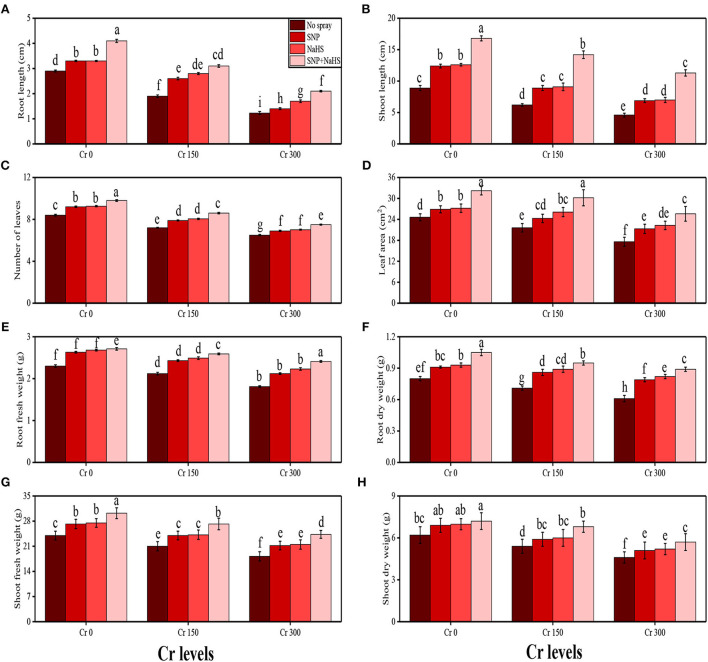
Impact of individual and combinatorial application of SNP and NaHS on growth attributes, i.e., **(A)** root length, **(B)** shoot length, **(C)** number of leaves, **(D)** leaf area, **(E)** root fresh weight, **(F)** root dry weight, **(G)** shoot fresh weight, and **(H)** shoot dry weight of spinach (*S. oleracea*) plants grown under different levels of Cr [0 (no Cr), 150, and 300 μM] in sandy loam soil. Values in the figures indicate just one harvest. Mean ± *SD* (*n* = 4). Two-way ANOVA was performed and mean differences were tested by HSD (*P* < 0.05). Different lowercase letters on the error bars indicate significant differences between the treatments.

**Figure 2:**
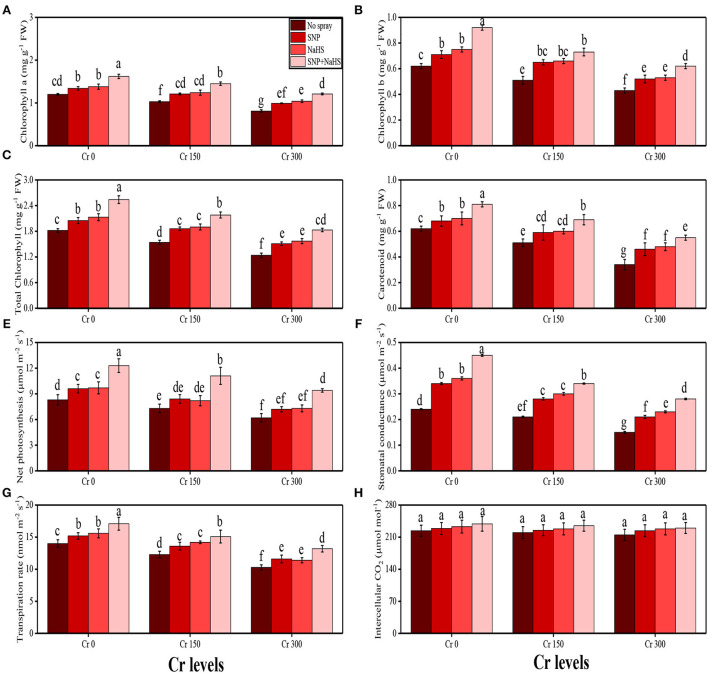
Impact of individual and combinatorial application of SNP and NaHS on photosynthetic pigments, i.e., chlorophyll-a content **(A)**, chlorophyll-b content **(B)**, total chlorophyll content **(C)**, carotenoid content **(D)**, net photosynthesis **(E)**, stomatal conductance **(F)**, transpiration rate **(G)**, and intercellular CO_2_ **(H)** of spinach (*S. oleracea*) plants grown under different levels of Cr [0 (no Cr), 150, and 300 μM] in sandy loam soil. Values in the figures indicate just one harvest. Mean ± *SD* (*n* = 4). Two-way ANOVA was performed and mean differences were tested by HSD (*P* < 0.05). Different lowercase letters on the error bars indicate significant differences between the treatments.

In the present study, various growth and photosynthetic parameters were also measured in *S. oleracea* grown under the different levels of Cr [0 (no Cr), 150, and 300 μM] in the soil which was also supplemented with different exogenous levels of SNP and NaHS. The data regarding shoot length, root length, number of leaves, leaf area, shoot fresh weight, root fresh weight, shoot dry weight, and root dry weight are presented in Figure 1, and the data regarding the content of chlorophyll-a, chlorophyll-b, total chlorophyll, and carotenoid, net photosynthesis, stomatal conductance, transpiration rate, and intercellular CO_2_ are presented in Figure 2. According to the results, it was noticed that the increasing levels of Cr in the soil significantly (*p* < 0.05) decreased plant growth and biomass and photosynthetic pigments in *S. oleracea* without the application of SNP and NaHS in the soil (Figures 1, 2). According to the given results, increasing levels of Cr (50 and 100 μM) in the soil significantly (*p* < 0.05) decreased shoot length, root length, number of leaves, leaf area, shoot fresh weight, root fresh weight, shoot dry weight, root dry weight, chlorophyll-a, chlorophyll-b, total chlorophyll, carotenoid content, net photosynthesis, stomatal conductance, and transpiration rate in *S. oleracea*, compared to the plants grown without the treatment of Cr in the soil. The exogenous application of SNP and NaHS was also performed to measure various growth (Figure 1) and photosynthetic attributes (Figure 2) in *S. oleracea* grown under the elevating levels of Cr in the soil. The application of Si non-significantly increased shoot length, root length, the number of leaves, leaf area, shoot fresh weight, root fresh weight, shoot dry weight, root dry weight, chlorophyll-a, chlorophyll-b, total chlorophyll, carotenoid content, net photosynthesis, stomatal conductance, and transpiration rate at all levels of Cr in the soil, compared to the plants which were grown without the application of SNP and NaHS. We also noticed that Cr toxicity did not significantly affect the intercellular CO_2_ levels and also application of SNP and NaHS did not significantly influence the intercellular CO_2_ levels in *S. oleracea* under all levels of As in the soil (Figure 2H).

### Impact of individual/or combinatorial application of SNP and NaHS on oxidative stress and antioxidant capacity under Cr stress

#### Oxidative stress indicators

**Figure 3:**
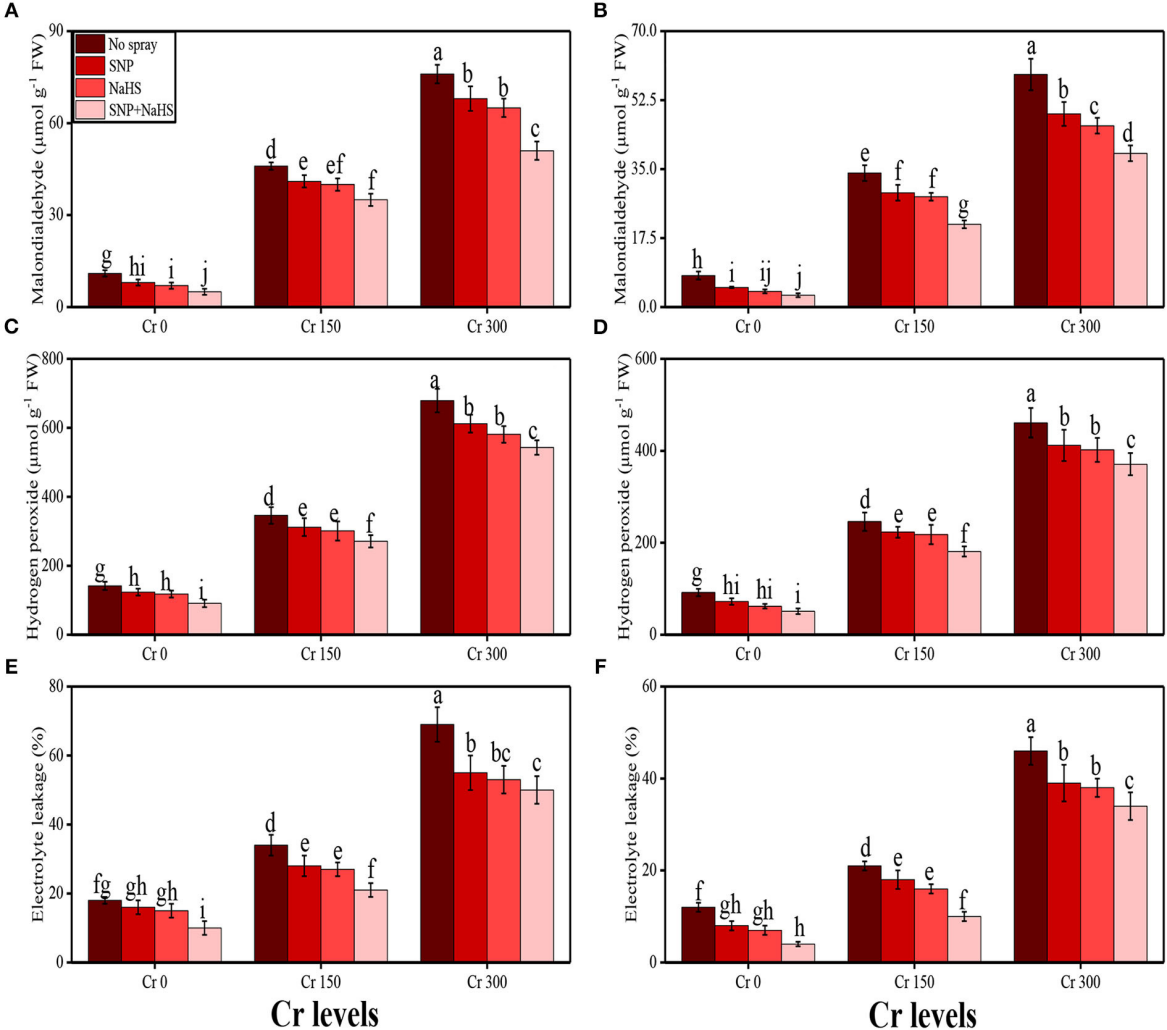
Impact of individual and combinatorial application of SNP and NaHS on oxidative stress indicators, i.e., MDA content in the roots **(A)**, MDA content in the leaves **(B)**, H_2_O_2_ content in the roots **(C)**, H_2_O_2_ content in the leaves **(D)**, EL percentage in the roots **(E)**, and EL percentage in the leaves **(F)** of spinach (*S. oleracea*) plants grown under different levels of Cr [0 (no Cr), 150, and 300 μM] in sandy loam soil. Values in the figures indicate just one harvest. Mean ± *SD* (*n* = 4). Two-way ANOVA was performed and mean differences were tested by HSD (*P* < 0.05). Different lowercase letters on the error bars indicate significant differences between the treatments.

Malondialdehyde (MDA) content, hydrogen peroxide (H_2_O_2_) initiation, and electrolyte leakage (%) increased in the roots and shoots of *S. oleracea* with the increasing concentrations of Cr (150 and 300 μM) in the soil medium without SNP and NaHS when compared to the plants grown in 0 mM Cr. The data regarding oxidative stress indicators in the roots and shoots of *S. oleracea* are presented in Figure 3. It was observed that the contents of MDA, H_2_O_2_, and EL (%) were increased in the roots and also in the shoots grown in 300 μM of Cr without the application of SNP and NaHS when compared to those plants grown in 0 μM of Cr in the soil without the application of SNP and NaHS. Application of SNP and NaHS significantly decreased the contents of MDA, H_2_O_2_, and EL (%) in the roots and also in the shoots of the plants grown with Cr level of 300 μM under SNP and NaHS application when compared to those plants grown with 300 μM Cr without the application of SNP and NaHS.

#### Antioxidant compounds

**Figure 4:**
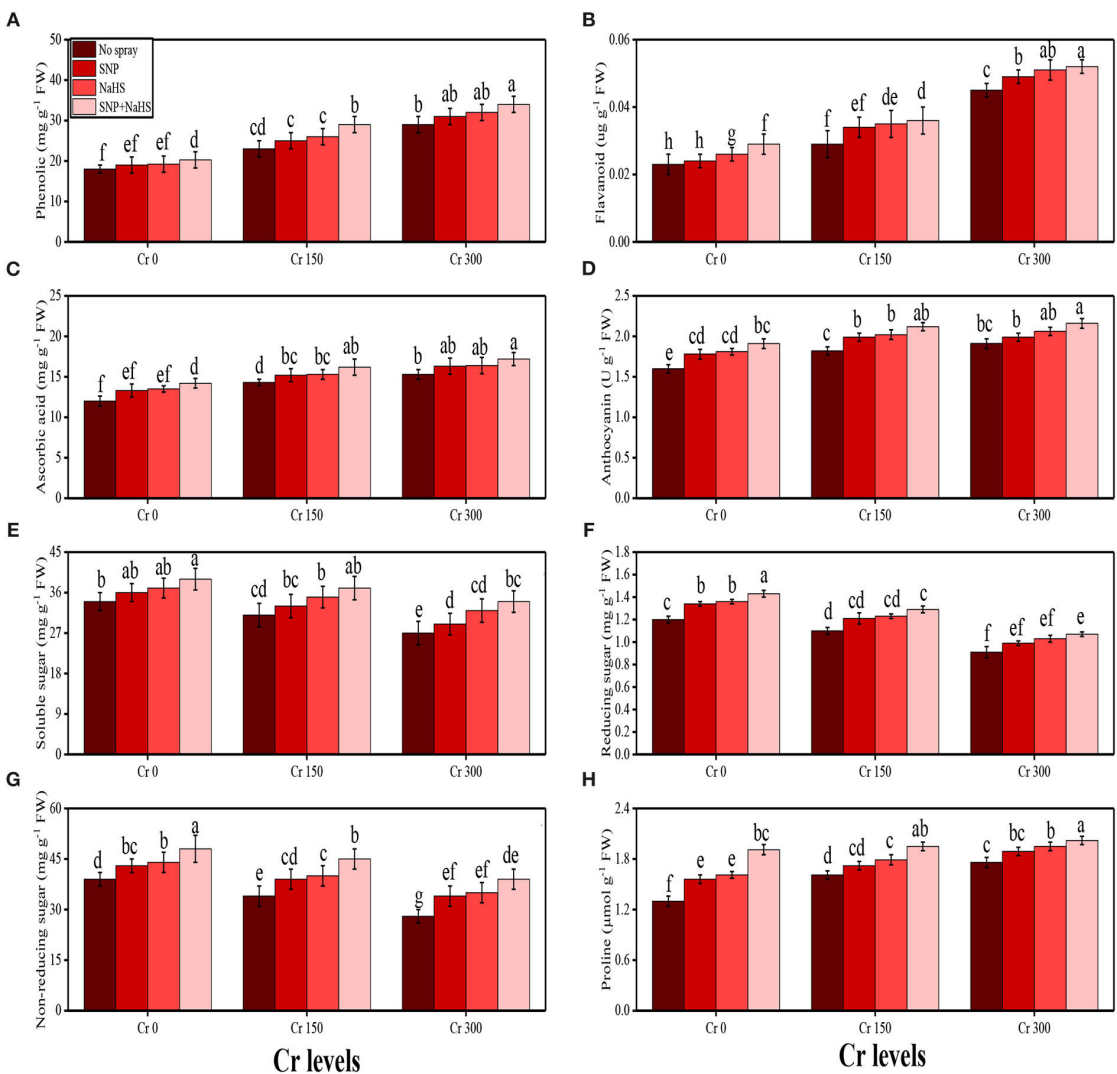
Impact of individual and combinatorial application of SNP and NaHS on osmolytes and sugars, i.e., phenolic content **(A)**, flavonoid content **(B)**, ascorbic acid content **(C)**, anthocyanin content **(D)**, soluble sugar content **(E)**, reducing sugar content **(F)**, non-reducing sugar content **(G)**, and proline content **(H)** of spinach (*S. oleracea*) plants grown under different levels of Cr [0 (no Cr), 150, and 300 μM] in sandy loam soil. Values in the figures indicate just one harvest. Mean ± *SD* (*n* = 4). Two-way ANOVA was performed and mean differences were tested by HSD (*P* < 0.05). Different lowercase letters on the error bars indicate significant differences between the treatments.

The activities of various antioxidant enzymes, such as superoxidase dismutase (SOD), peroxidase (POD), catalase (CAT), and ascorbate peroxidase (APX), in the roots and shoots of *S. oleracea* seedlings and the content of non-enzymatic compounds, such as phenolic, flavonoid, ascorbic acid, and anthocyanin, were also measured in the present study. The data regarding the activities of enzymatic antioxidants (SOD, POD, CAT, and APX) are presented in Figure 3, and the results regarding the content of non-enzymatic antioxidants (phenolic, flavonoid, ascorbic acid, and anthocyanin) are presented in Figure 4. The results showed that the activities of enzymatic antioxidants (SOD, POD, CAT, and APX) and the content of non-enzymatic antioxidants (phenolic, flavonoid, ascorbic acid, and anthocyanin) were increased up to a Cr level of 300 μM in the soil. The results also showed that the addition of SNP and NaHS non-significantly increased the activities of enzymatic antioxidants (SOD, POD, CAT, and APX) and the content of non-enzymatic antioxidants (phenolic, flavonoid, ascorbic acid, and anthocyanin) at all levels of Cr [0 (no Cr), 150, and 300 μM] in the soil, compared to the plants that were grown in the soil not supplemented with SNP and NaHS.

#### Sugar, proline, and nutrient uptake by the plant parts

**Figure 5:**
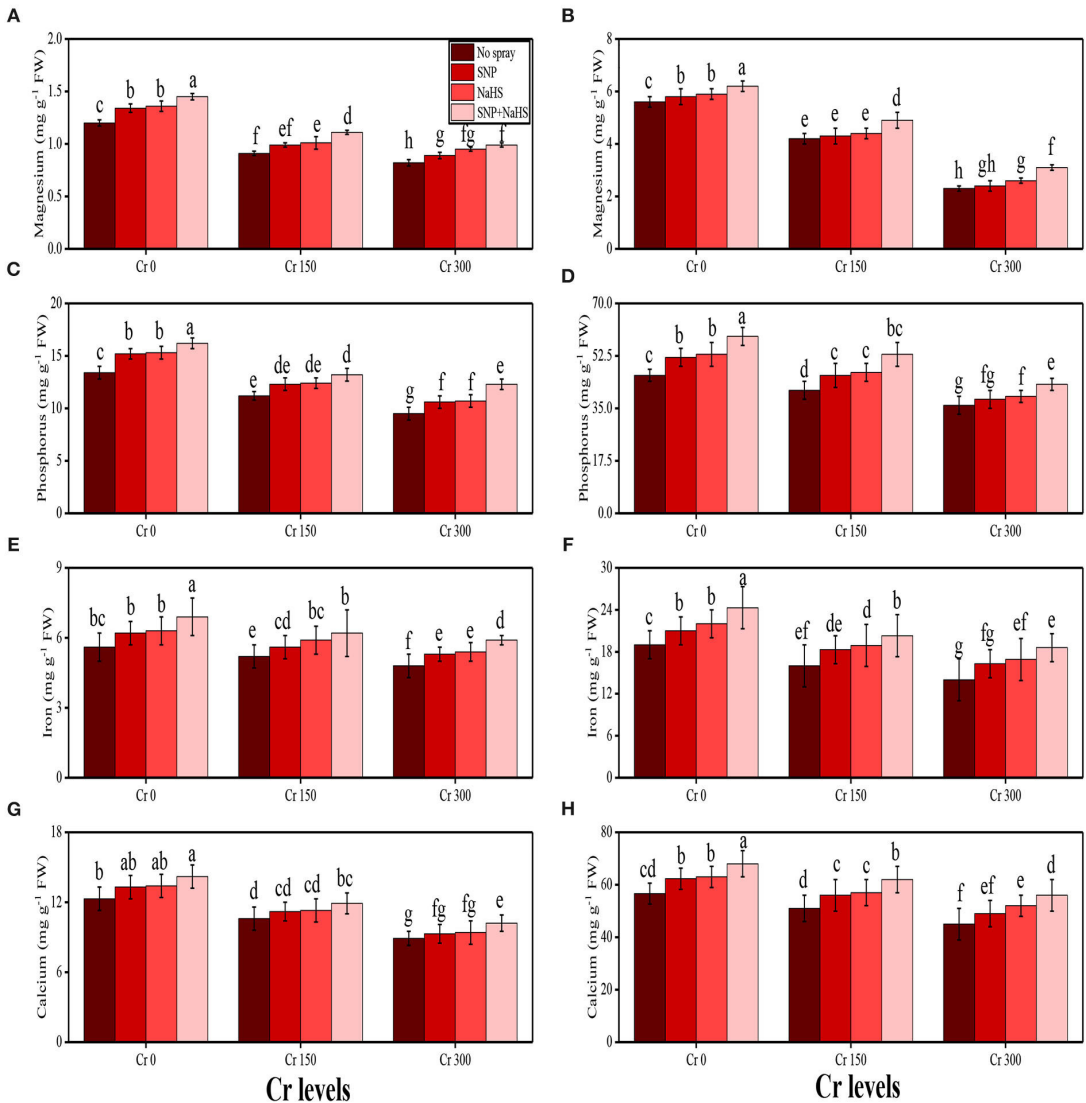
Impact of individual and combinatorial application of SNP and NaHS on nutritional status, i.e., magnesium content in the roots **(A)**, magnesium content in the shoots **(B)**, phosphorus content in the roots **(C)**, phosphorus content in the shoots **(D)**, iron content in the roots **(E)**, iron content in the shoots **(F)**, calcium content in the roots **(G)**, and calcium content in the shoots **(H)** of spinach (*S. oleracea*) plants grown under different levels of Cr [0 (no Cr), 150, and 300 μM] in sandy loam soil. Values in the figures indicate just one harvest. Mean ± *SD* (*n* = 4). Two-way ANOVA was performed and mean differences were tested by HSD (*P* < 0.05). Different lowercase letters on the error bars indicate significant differences between the treatments.

The content of soluble sugar, reducing sugar, non-reducing sugar, proline, and various nutrients, such as calcium (Ca^2+^), magnesium (Mg^2+^), iron (Fe^2+^), and phosphorus (P), were also measured in the roots and shoots of *S. oleracea* in the present study under the different levels of Cr [0 (no Cr), 150, and 300 μM] in the soil which was also supplemented with SNP and NaHS. The data regarding the content of soluble sugar, reducing sugar, non-reducing sugar, and proline are presented in Figure 5, and the data regarding the content of Ca^2+^, Mg^2+^, Fe^2+^, and P in the roots and shoots of the plants are presented in Figure 5. The results of the present study show that the increasing levels of Cr in the soil significantly (*P* < 0.05) decreased the content of nutrients (Ca^2+^, Mg^2+^, Fe^2+^, and P) in the roots and shoots of the plants and also decreased the content of sugars (soluble sugars, reducing sugars, and non-reducing sugars), compared to the plants that were grown in the soil which was not treated with Cr. However, the content of proline was increased by increasing the levels of Cr in the soil, when compared to the plants which were not treated with Cr (Figure 4H). The content of various sugars (Figure 4), phenolic acids, and nutrients (Figure 5) in the shoots of the plants was determined after the application of SNP and NaHS to the soil The results suggested that the application of SNP and NaHS non-significantly increased the sugar content (soluble sugars, reducing sugars, and non-reducing sugars) and proline content in the shoots and significantly increased the content of nutrients (Ca^2+^, Mg^2+^, Fe^2+^, and P) in the roots and shoots of the plants, compared to the plants grown without the treatment of SNP and NaHS at all the levels of Cr in the soil.

### Impact of individual /or combinatorial application of SNP and NaHS on organic acid exudation and Cr uptake under Cr stress

**Figure 6:**
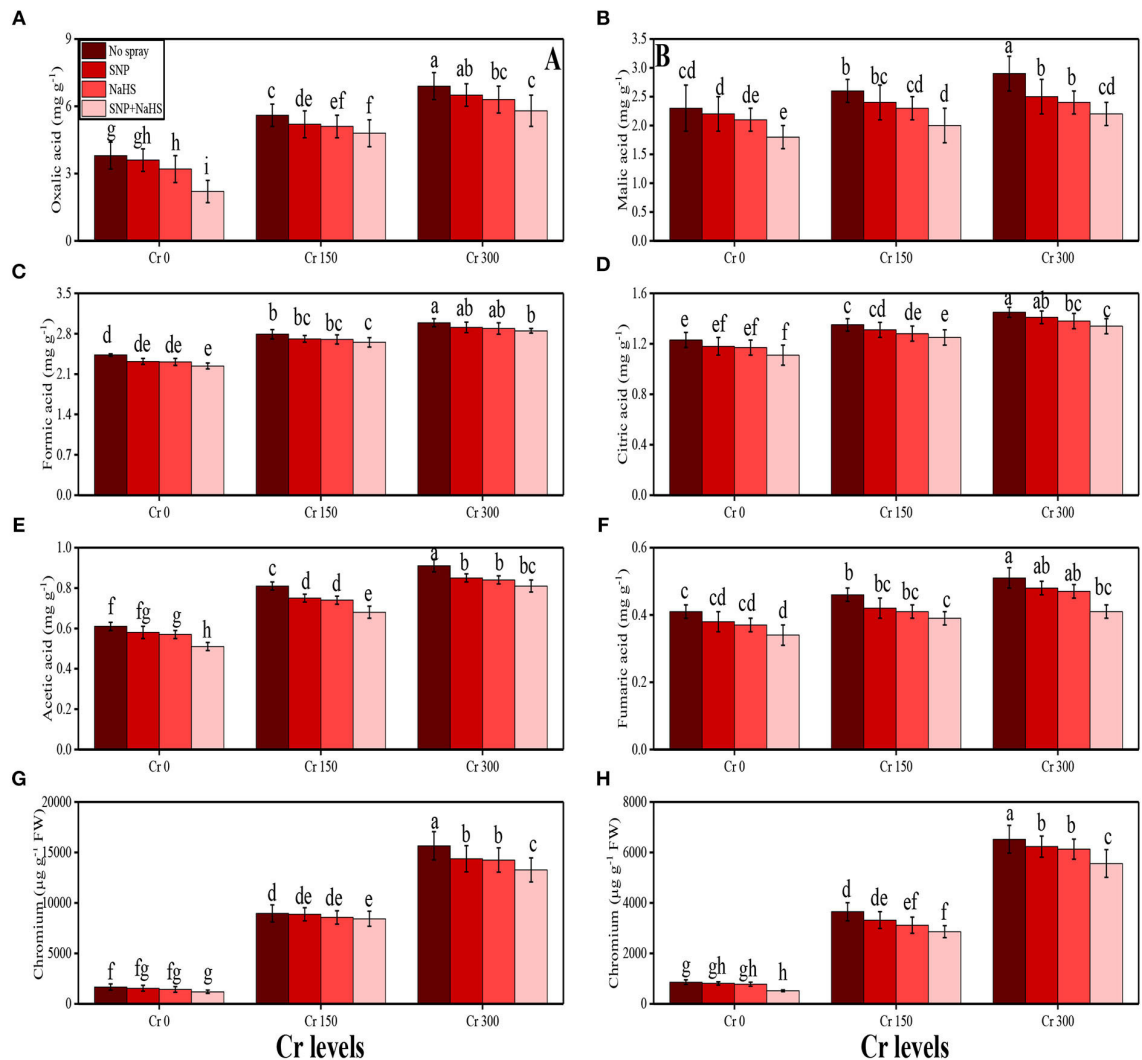
Impact of individual and combinatorial application of SNP and NaHS on organic acids and Co uptake, i.e., oxalic acid content **(A)**, malic acid content **(B)**, formic acid content **(C)**, citric acid content **(D)**, acetic acid content **(E)**, and fumaric acid content **(F)** in the roots, Cr content in the roots **(G)**, and Cr content in the shoots **(H)** of spinach (*S. oleracea*) plants grown under different levels of Cr [0 (no Cr), 150, and 300 μM] in sandy loam soil. Values in the figures indicate just one harvest. Mean ± *SD* (*n* = 4). Two-way ANOVA was performed and mean differences were tested by HSD (*P* < 0.05). Different lowercase letters on the error bars indicate significant differences between the treatments.

**Figure 7:**
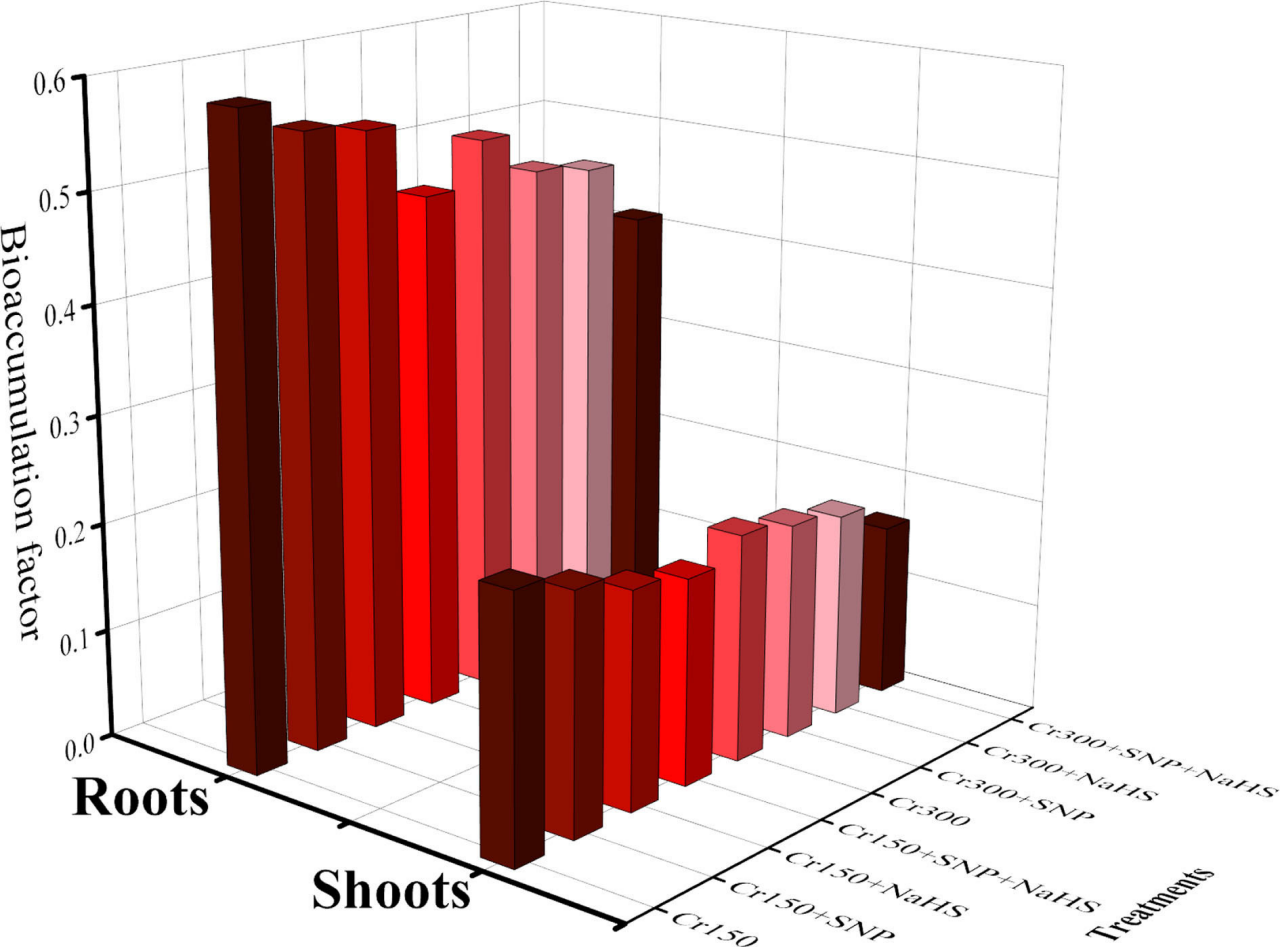
Bioaccumulation factor of roots and shoots of spinach (*S. oleracea*) plants grown under the different levels of Cr stress, i.e., 150 and 300 μM, in sandy loam soil with alone/or combinatorial application of SNP and NaHS.

The content of fumaric acid, formic acid, acetic acid, citric acid, malic acid, and oxalic acid in the roots and Cr concentration in the roots and shoots of *S. oleracea* grown under toxic levels of Cr in the soil, with or without the application of SNP and NaHS, are presented in Figure 6. According to the given results, we have noticed that elevated concentrations of Cr in the soil (150 and 300 μM) induced a significant (*P* < 0.05) increase in the content of fumaric acid, formic acid, acetic acid, citric acid, malic acid, and oxalic acid in the roots and also Cr concentration in the roots and shoots of *S. oleracea*, compared to the plants that were grown in the soil treated with 0 μM Cr. The results also illustrated that the application of SNP and NaHS decreased the content of fumaric acid, formic acid, acetic acid, citric acid, malic acid, and oxalic acid in the roots and also Cr concentration in the roots and shoots of *S. oleracea*, compared with the plants that were grown without the exogenous application of SNP and NaHS in the soil. We also presented the data regarding the bioaccumulation factor (BAF) of Cr in the roots and shoots of *S. oleracea* in Figure 7. All the values of BAF were <1, which indicates that the addition of SNP and NaHS (combined or alone) decreased the values of BAF in the roots and shoots of *S. oleracea*. In addition, at all levels of Cr stress (150 and 300 μM), the content of fumaric acid, formic acid, acetic acid, citric acid, malic acid, and oxalic acid and also As concentration in the roots and shoots decreased with the application of SNP and NaHS in the soil, compared with the plants that were grown without the application of SNP and NaHS.

### Correlation between Cr uptake and different morpho-physio attributes of *S. oleracea*

**Figure 8:**
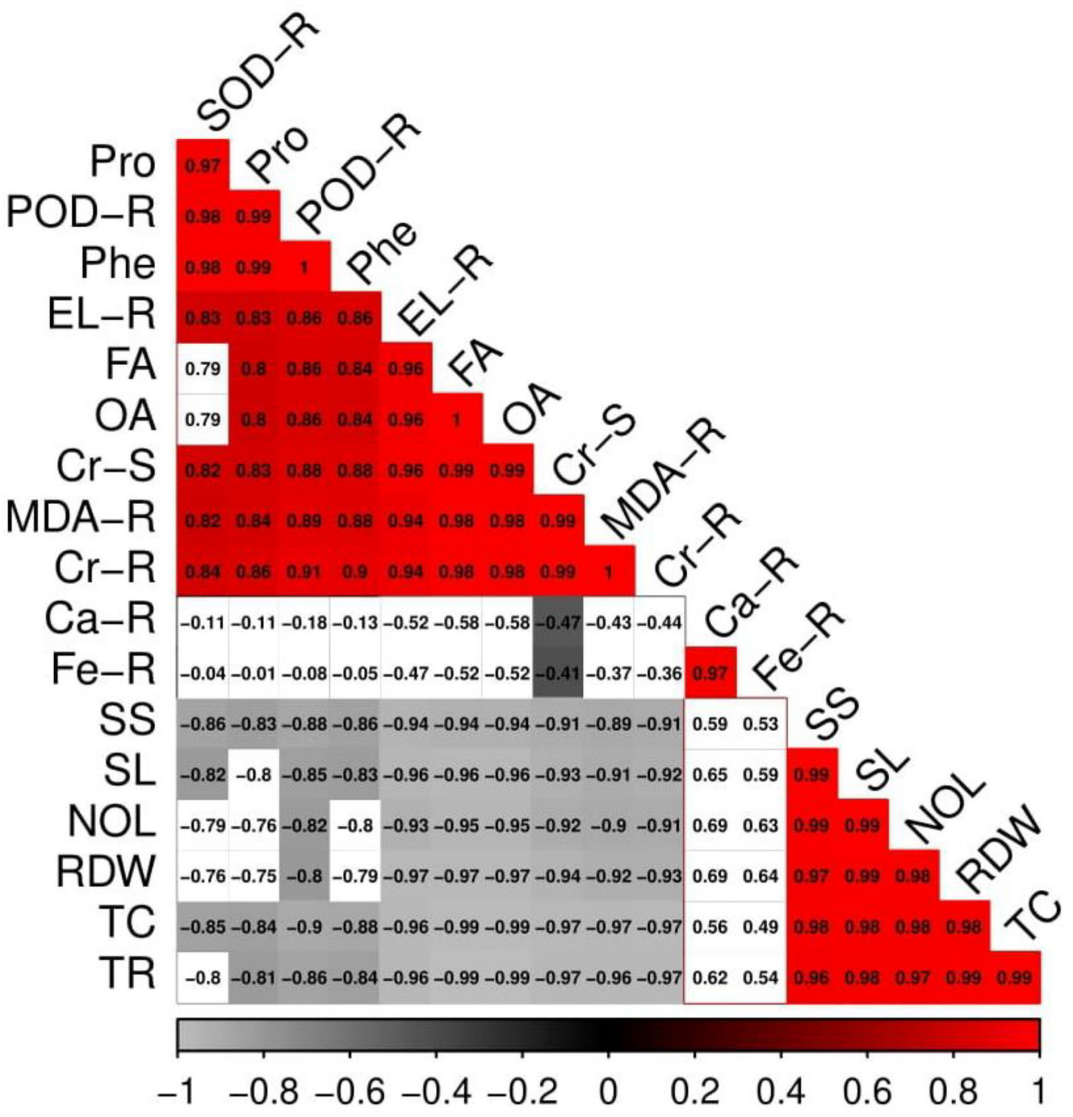
Correlation between Cr uptake in different parts of the plant and some selected traits of morphological attributes, photosynthetic efficiency, oxidative stress and response of antioxidant enzymes, nutrient uptake, and organic acid exudation pattern. Different abbreviations used in the figure are as follows: SOD-R, superoxidase dismutase activity in the roots; Pro, proline content; POD-R, peroxidase activity in the roots; Phe, phenolic content; EL-R, electrolyte leakage in the roots; FA, fumaric acid content; OA, oxalic acid content; Cr-S, chromium content in the shoots; MDA-R, malondialdehyde content in the roots; Cr-R, chromium content in the roots; Ca-R, calcium content in the roots; Fe-R, iron content in the roots; SS, soluble sugar content; SL, shoot length; NOL, number of leaves; RDW, root dry weight; TC, total chlorophyll content; TR, transpiration rate.

A Pearson's correlation graph was constructed to quantify the relationship between various growth parameters and Cr uptake in different parts of the plant (Figure 8). Cr concentration in the roots was positively correlated with Cr concentration in the shoots, superoxidase dismutase, peroxidase, proline, electrolyte leakage, fumaric acid, oxalic acid, malondialdehyde, calcium content in the roots, and iron content in the roots, while negatively correlated with soluble sugar, shoot length, root dry weight, number of leaves, total chlorophyll content, and transpiration rate. Similarly, Cr concentration in the shoots was positively correlated with Cr concentration in the roots, superoxidase dismutase, peroxidase, proline, electrolyte leakage, fumaric acid, oxalic acid, malondialdehyde, calcium content in the roots, and iron content in the roots, while negatively correlated with soluble sugar, shoot length, root dry weight, number of leaves, total chlorophyll content, and transpiration rate. This relationship showed a close association between plant composition and Cr accumulation in various parts of the plant.

## Discussion

A rapid rise in population coupled with industrialization and urbanization has led to environmental pollution. Heavy metals are hazardous pollutants present in untreated industrial effluents, which can negatively affect the environment and health of human beings (Zaheer et al., 2020b; Javed et al., 2021; Saleem et al., 2022b). The enhanced accretion of Cr in farming lands causes a reduction in the productivity of economically significant crops (Ali et al., 2013; Zaheer et al., 2020a). Cr-induced phytotoxicity can be attributed to the production of free radicals, which enhance the degradation of biomolecules present in the cells of plants (Ali et al., 2013; Danish et al., 2019). Cr treatment showed cytotoxic and genotoxic effects and hormonal imbalance, which further affects the germination and development of plants and reduces dry matter production (Hussain et al., 2018; Maqbool et al., 2018). Similar results were observed in the present study, which showed that increasing levels of Cr in the soil (150 and 300 μM) decreased plant growth and biomass (Figure 1) and also decreased the photosynthetic pigments and gas exchange characteristics (Figure 2). Excessive Cr concentrations negatively impact photosynthesis by affecting the enzymes of Calvin cycle, thylakoid membrane, and photosynthetic electron transport. The presence of Cr showed a reduction in pigment biosynthesis; inhibition of net photosynthetic rate, stomatal conductance, electron transport chain, fixation of carbon dioxide, and photosynthetic phosphorylation; affects the plastid structure and function; and negatively affects light/dark reactions (Tripathi et al., 2012; Ashraf et al., 2022).

Plants being sessile organisms are often exposed to heavy metal stress, as they have no choice to escape from unfavorable environmental conditions (Rana et al., 2020; Saleem et al., 2020a; Mehmood et al., 2021). Under Cr exposure, plants suffer through morphological biochemical alterations because of the imbalance between the production and removal of free radicals, also known as an oxidative burst (Tripathi et al., 2012; Gautam et al., 2020). Free radicals are generated in different organelles, such as chloroplast, peroxisome, and mitochondria, as a by-product of different biochemical processes. Escalated generation of ROS in plants under Cr toxicity promotes damage to pigments, nucleic acids, and proteins and increases lipid peroxidation (Qureshi et al., 2020; Usman et al., 2020). Adverse impacts on various morphological and physiological processes have been reported in the present study (Figure 8), where Cr enhanced the degradation of plasma membrane permeability by the generation of oxidative stress indicators (Ma et al., 2020; Zainab et al., 2021). Plants with an effective antioxidant system are able to control high concentrations of Cr (Figure 3). In addition, Cr stress-induced decrease in photosynthesis in plants may also be due to the overproduction of ROS in the soil through oxidation/reduction mechanisms (Ugwu and Agunwamba, 2020), which ultimately leads to the induction of oxidative stress and reduction of plant growth, biomass, and yield (Vernay et al., 2007; Farid et al., 2019). However, the expression of antioxidative enzymes, such as SOD, POD, CAT, and APX, under a Cr-stressed environment plays a significant role in reducing Cr toxicity, which was reported in a number of studies conducted on various plant species (Usman et al., 2020; Zaheer et al., 2020c). Plants produce a variety of secondary metabolites, such as proline, flavonoids, and phenolics, that improve tolerance against metal toxicity. Although proline accumulation in plant tissue/organs is a response to metal toxicity, it might be associated with signal transduction and plays a role in preventing membrane distortion, which has been observed in many plant species (Saleem et al., 2020b; Mumtaz et al., 2021).

Since Cr has a close structural resemblance to many of the essential elements, it can influence the mineral nutrient status in plants (Dube et al., 2003; Ali et al., 2018). Cr is a non-essential element; hence, the plant body has no specific mechanism for its uptake (Gardea-Torresdey et al., 2004; Tiwari et al., 2013). Having a structure similar to many essential nutrients, Cr may interfere with the uptake, translocation, and accumulation of these nutrients (Dube et al., 2003; Ulhassan et al., 2019; Zaheer et al., 2020c). It easily interrupts the uptake of the essential elements required for the plant life cycle (Sundaramoorthy et al., 2010). Generally, nutrient availability also depends on root structure and the concentration of elements in the medium (González-Pérez et al., 2004; Chandrasekhar and Ray, 2017). Tiwari et al. (2013) reported that Cr affects the plant metabolism by interfering with the essential nutrients or by activating enzymes at the functional sites. Excessive Cr levels reduced the uptake of essential minerals by masking the sorption sites via the formation of insoluble complexes. A reduction in nutrient uptake/translocation was reported in the presence of Cr due to the competitive binding of Cr by transport channels and decreased plasma membrane H^+^ ATPase activity (Shahid et al., 2017). Under Cr toxicity, impairment of root growth and depletion in root penetration capacity were responsible for the decline in the absorption and transport of nutrients. The roots of plants secrete various organic acids, which act as ligands and can change insoluble metals present in the soil into soluble forms. Cr is actively taken up by the plasma membrane by involving phosphate and sulfate transporters, whereas trivalent Cr enters via cation exchange sites present in the plant cell wall (Singh et al., 2013; Ugwu and Agunwamba, 2020). Cr dispensation and translocation in plants depend on the oxidation state of Cr ions, its amount in the nutrient medium, and the species of plants.

**Figure 9:**
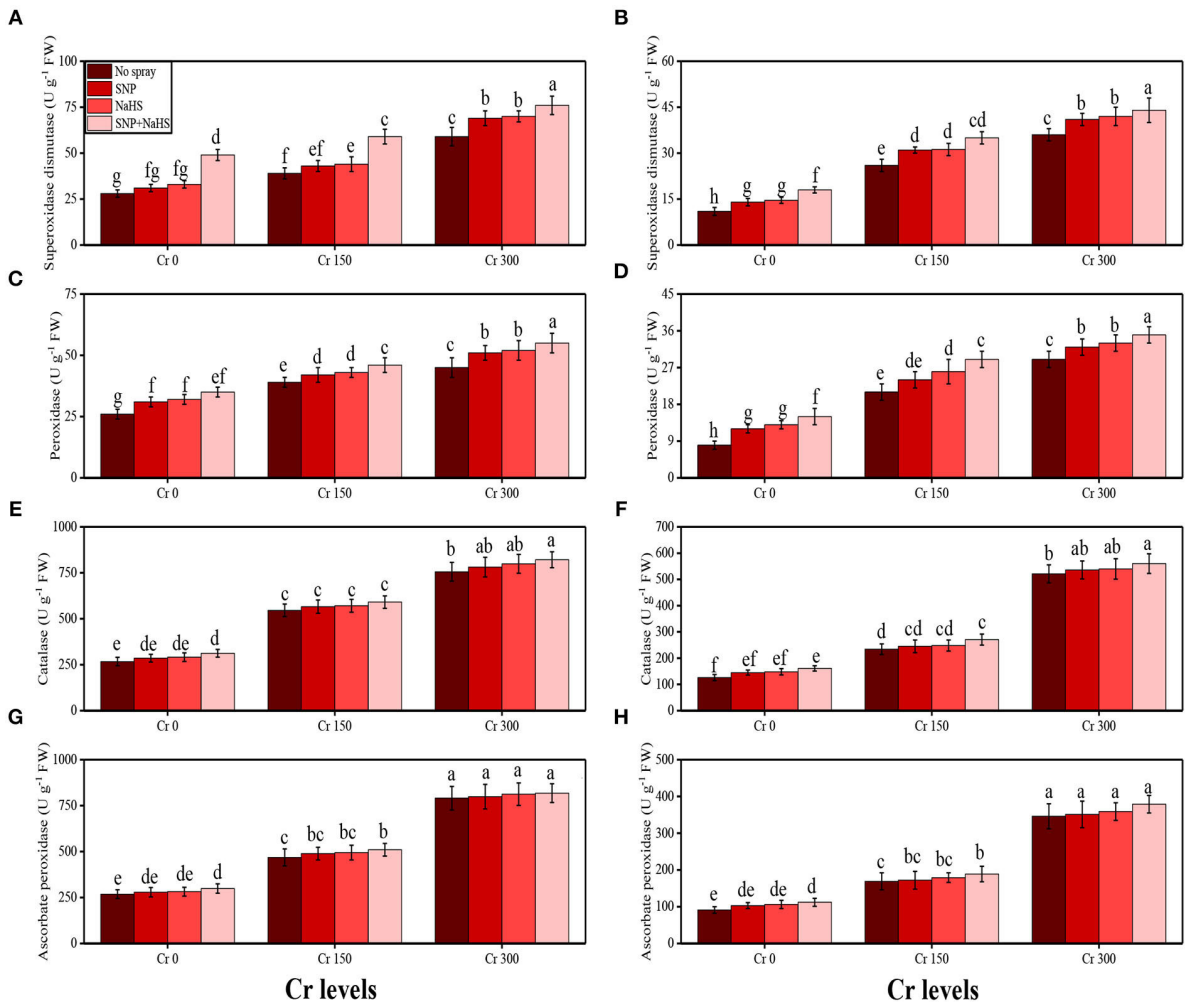
Impact of individual and combinatorial application of SNP and NaHS on enzymatic antioxidative enzymes, i.e., SOD activity in the roots **(A)**, SOD activity in the leaves **(B)**, POD activity in the roots **(C)**, POD activity in the leaves **(D)**, CAT activity in the roots **(E)**, CAT activity in the leaves **(F)**, APX activity in the roots **(G)**, and APX activity in the leaves **(H)** of spinach (*S. oleracea*) plants grown under different levels of Cr [0 (no Cr), 150, and 300 μM] in sandy loam soil. Values in the figures indicate just one harvest. Mean ± *SD* (*n* = 4). Two-way ANOVA was performed and mean differences were tested by HSD (*P* < 0.05). Different lowercase letters on the error bars indicate significant differences between the treatments.

The SNP acts as an essential signaling molecule that alleviates inhibitory effects of biotic and abiotic stresses on plant growth and development (Kaya et al., 2020b; Ali et al., 2022a). The SNP-induced increase in plant growth (Figure 1) and photosynthetic pigments (Figure 2) could be attributed to the involvement of SNP in the regulation of nutrient uptake (Figure 4) and plant growth (Gill et al., 2013; Arora and Bhatla, 2017). Furthermore, SNP can relax the cell wall, influencing phospholipid bilayers, increase the fluidity of cell membranes, and induce cell enlargement (Kaya et al., 2019; Ozfidan-Konakci et al., 2020; Pirooz et al., 2021). Moreover, SNP application also enhances the activity of antioxidant enzymes (Figure 3) and non-enzymatic compounds (Figure 9) that enhance plant growth under abiotic stress conditions (Rizwan et al., 2018; Habib et al., 2020). However, the application of SNP declined the Cr uptake in the roots and shoots of *S. oleracea* (Figure 5) and also the bioaccumulation factor (Figure 6), though SNP treatments more proficiently limited the Cr uptake or accumulation possibly via improving the nutrient balance (Ca^2+^, Mg^2+^, Fe^2+^, and P) (Figure 4). Earlier research studies on heavy metals also supported our outcomes that SNP can decrease the absorption and translocation of Co (Ozfidan-Konakci et al., 2020), Cd (Gill et al., 2013), and Cu (Pirooz et al., 2021). These authors suggested that SNP increases the biosynthesis of phytochelatins and the subsequent Cr-chelation and sequestration mechanism in roots under metal-stressed environments.

**Figure 10:**
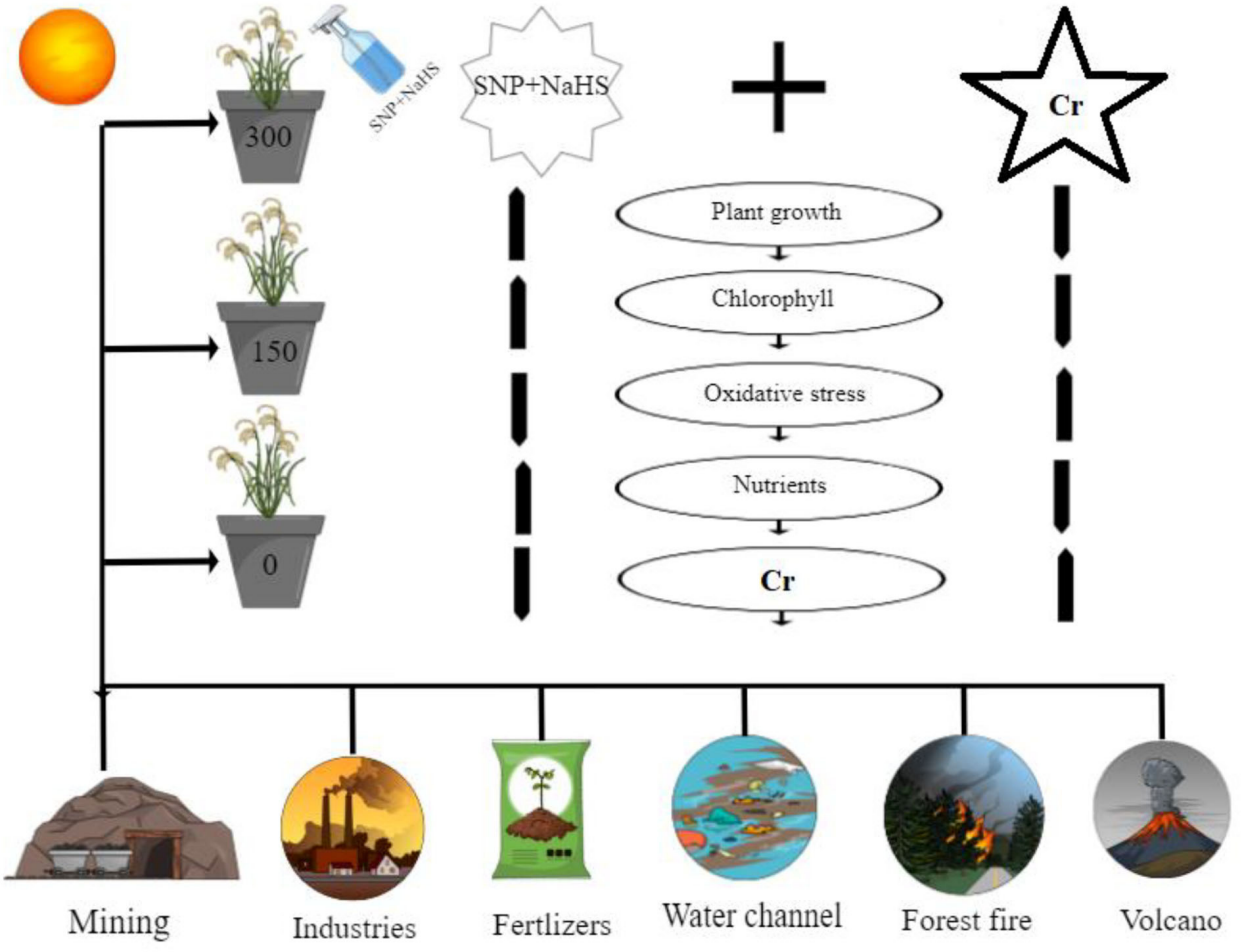
Schematic presentation of the findings from this study under the individual/or combinatorial application of SNP and NaHS in Cr-stressed spinach (*S. oleracea*) plants grown under different levels of Cr stress, i.e., 150 and 300 μM, in sandy loam soil. The figure shows Cr sources in the natural soil and its toxic effects on the plants. The figure also shows that Cr toxicity can be overcome by the alone/or combinatorial application of SNP and NaHS, which decreased oxidative stress in membrane-bound organelles by decreasing Cr content in various parts of the plants. Overall, this scheme presents the complete description of this experiment and the important findings that we have evaluated from the application of SNP and NaHS in Cr-stressed spinach (*S. oleracea*) plants.

Sodium hydrogen sulfide is known as an excellent signaling molecule that regulates various physicochemical processes in plants (Mfarrej et al., 2021). Exogenously applied NaHS can increase plant growth (Figure 1) by improving chlorophyll content (Figure 2). Increases in plant growth after NaHS application could be related to increased antioxidant capacity and reduced ROS accumulation (Kaya et al., 2018, 2020a). However, inhibition of plant growth and reduction in chlorophyll content and photosynthetic parameters were greatly alleviated by exogenous application of NaHS (Figures 1, 2). NaHS has been reported to be the third gas transmitter after NO and CO in animals (Ali et al., 2014), but there is still little evidence demonstrating the role of H_2_S as a signal molecule in plants. Many studies have shown that sulfur-containing defense compounds, which include elemental sulfur (S^0^), H_2_S, glutathione, phytochelatins, various secondary metabolites, and sulfur-rich proteins, are crucial for the survival of plants under abiotic stresses. However, whether volatile NaHS belongs to the group of sulfur-containing defense compounds is still controversial. The involvement of NaHS in the alleviation of Pb (Ali et al., 2014), osmotic (Zhang et al., 2010), Al (Chen et al., 2013), and Cr (Ahmad et al., 2019) stress has recently been reported. The schematic presentation of the mechanistic role of SNP and NaHS in alleviating the Cr toxicity in *S. oleracea* is presented in Figure 10.

## Conclusion

On the basis of these findings, it can be concluded that the negative impact of Cr toxicity can be overcome by the external application of SNP and NaHS (individually or in combination). Our results depict that Cr toxicity induced severe metal toxicity in *S. oleracea* by increasing the generation of ROS in the form of oxidative stress and also increasing the concentration of Cr in the roots and shoots of the plants. Furthermore, Cr toxicity also increased organic acid exudation and imbalance in the nutritional status of the plants, which ultimately decrease plant growth, yield, and photosynthetic efficiency. Hence, Cr toxicity was eliminated by the external application of SNP and NaHS, which also decreased the Cr concentration in the plant tissues, degenerated ROS, and organic acid exudation, but increased the activities of antioxidants and essential nutrients in the plants. Therefore, long-term field studies should be executed to draw parallels amongst plants/crops root exudations, metal stress, nutrient fertigation regimes, nutrient mobility patterns, and plant growth in order to gain insights into the underlying mechanisms.

## Data availability statement

The raw data supporting the conclusions of this article will be made available by the authors, without undue reservation.

## Author contributions

Conceptualization: IH and SE. Data curation: IH and SM. Formal analysis: FC, IH, DV, and SM. Funding acquisition: SE. Investigation: SM. Methodology: IH. Project administration: SA, AA, SM, and RM. Resources: FC, RM, and DV. Software: SA, AA, MS, JM, and DV. Supervision: SE, JM, and MS. Writing—original draft: SE, JM, RM, and MS. Writing—review and editing: MS, FC, SA, AA, JM, and RM. All authors contributed to the article and approved the submitted version.

## Funding

This publication was supported by funds from the National Research Development Projects to finance excellence (PFE)-14/2022-2024 granted by the Romanian Ministry of Research and Innovation and CASEE Fund for Incentives, project No: CASEE fund 2021-2. The authors extend their appreciation to the Deanship of Scientific Research at King Khalid University for funding this work through Research Group Project under grant number (R.G.P 1- 100/43). This work is also supported by National Natural Science Foundation of China grant no. (51974313).

## Conflict of interest

The authors declare that the research was conducted in the absence of any commercial or financial relationships that could be construed as a potential conflicts of interest.

## Publisher's note

All claims expressed in this article are solely those of the authors and do not necessarily represent those of their affiliated organizations, or those of the publisher, the editors and the reviewers. Any product that may be evaluated in this article, or claim that may be made by its manufacturer, is not guaranteed or endorsed by the publisher.
