# Supplementary material for: Individual and combinatorial effects of SNP and NaHS on morpho-physio-biochemical attributes and phytoextraction of chromium through Cr-stressed spinach (Spinacia oleracea L.)
Source: Front Plant Sci. 2022 Aug 17;13:973740. doi: 10.3389/fpls.2022.973740 (PMC9428630; doi:10.3389/fpls.2022.973740)
Supplement: Supplementary file 1 [file Table_1.DOCX]

**Table S1** Physical and nutritional properties of the soil used in this experiment

| ­­­­­­­**Physical properties** |  |
| --- | --- |
| Organic matter | >70% of total solids |
| Density | 350 Kg/m^3^ |
| pH | 7.6 |
| Electrical conductivity | 20mS/m |
| Organic nitrogen | 1400 mg/L |
| **Nutrients** | **g/m^3^** |
| Nitrogen (NO_3_-N + NH_4_-N) | 150 |
| Phosphorus (P) | 75 |
| Potassium (K) | 160 |
| Magnesium (Mg) | 250 |
| Calcium (Ca) | 1600 |
| Sulphur (S) | 85 |
| Copper (Cu) | 2.5 |
| Zinc (Zn) | 1.8 |
| Molybdenum (Mo) | 2.7 |
| Iron (Fe) | 5.6 |
